# Red fox viromes in urban and rural landscapes

**DOI:** 10.1093/ve/veaa065

**Published:** 2020-08-25

**Authors:** Sarah J Campbell, Wilbur Ashley, Margarita Gil-Fernandez, Thomas M Newsome, Francesca Di Giallonardo, Ayda Susana Ortiz-Baez, Jackie E Mahar, Alison L Towerton, Michael Gillings, Edward C Holmes, Alexandra J R Carthey, Jemma L Geoghegan

**Affiliations:** Department of Biological Sciences, Macquarie University, Sydney, New South Wales 2109, Australia; Department of Biological Sciences, Macquarie University, Sydney, New South Wales 2109, Australia; Department of Biological Sciences, Macquarie University, Sydney, New South Wales 2109, Australia; School of Life and Environmental Sciences, The University of Sydney, Sydney, New South Wales 2006, Australia; The Kirby Institute, University of New South Wales, Sydney, New South Wales 2052, Australia; Marie Bashir Institute for Infectious Diseases and Biosecurity, School of Life and Environmental Sciences and School of Medical Sciences, The University of Sydney, Sydney, New South Wales 2006, Australia; Marie Bashir Institute for Infectious Diseases and Biosecurity, School of Life and Environmental Sciences and School of Medical Sciences, The University of Sydney, Sydney, New South Wales 2006, Australia; Greater Sydney Local Land Services, Sydney, New South Wales 2750, Australia; Department of Biological Sciences, Macquarie University, Sydney, New South Wales 2109, Australia; Marie Bashir Institute for Infectious Diseases and Biosecurity, School of Life and Environmental Sciences and School of Medical Sciences, The University of Sydney, Sydney, New South Wales 2006, Australia; Department of Biological Sciences, Macquarie University, Sydney, New South Wales 2109, Australia; Department of Biological Sciences, Macquarie University, Sydney, New South Wales 2109, Australia; Department of Microbiology and Immunology, University of Otago, Dunedin 9016, New Zealand; Institute of Environmental Science and Research, Wellington 5018, New Zealand

**Keywords:** Vulpes vulpes, carnivore, predator, canine, exotic species, urban, virus, metagenomics

## Abstract

The Red fox (*Vulpes vulpes*) has established large populations in Australia’s urban and rural areas since its introduction following European settlement. The cryptic and highly adaptable nature of foxes allows them to invade cities and live among humans whilst remaining largely unnoticed. Urban living and access to anthropogenic food resources also influence fox ecology. Urban foxes grow larger, live at higher densities, and are more social than their rural counterparts. These ecological changes in urban red foxes are likely to impact the pathogens that they harbour, and foxes could pose a disease risk to humans and other species that share these urban spaces. To investigate this possibility, we used a meta-transcriptomic approach to characterise the virome of urban and rural foxes across the Greater Sydney region in Australia. Urban and rural foxes differed significantly in virome composition, with rural foxes harbouring a greater abundance of viruses compared to their urban counterparts. We identified ten potentially novel vertebrate-associated viruses in both urban and rural foxes, some of which are related to viruses associated with disease in domestic species and humans. These included members of the *Astroviridae*, *Picobirnaviridae*, *Hepeviridae*, and *Picornaviridae* as well as rabbit haemorrhagic disease virus-2. This study sheds light on the viruses carried by urban and rural foxes and emphasises the need for greater genomic surveillance of foxes and other invasive species at the human–wildlife interface.

## 1. Introduction

Red foxes (*Vulpes vulpes*) have the largest natural distribution of any wild terrestrial carnivore (Schipper et al. 2008), extending through Eurasia and North America (Statham et al. 2014). Introduced to Australia in the mid-1800s, they rapidly expanded across the continent. Red foxes exploit a wide range of habitats with varying climates, from alpine to desert, and are considered one of the most adaptable species on the planet. They are broadly distributed across natural and forested landscapes as well as highly urbanised, human dominated areas (Saunders, Gentle, and Dickman 2010; Bateman and Fleming 2012). Red fox home ranges vary depending on resource availability and land-use type. In Australia, home ranges for foxes in arid regions can reach at least 120 km^2^ (Newsome, Spencer, and Dickman 2017), between 5 and 7km^2^ in rural areas (Coman, Robinson, and Beaumont 1991) and <1 km^2^ in urban centres (Marks and Bloomfield 2006).

Foxes have recently established a large presence in major metropolitan centres (Marks and Bloomfield 1999; Saunders, Gentle, and Dickman 2010). Urban areas support surprisingly high densities of foxes. For example, there are up to sixteen individuals per km^2^ in Melbourne (Marks and Bloomfield 1999), compared to just 0.2 individuals per km^2^ in rural areas (Saunders, Gentle, and Dickman 2010). In Bristol city in the UK, densities reach as high as thirty-five foxes per km^2^ (Baker et al. 2001).

Predation by red foxes is a key threat to Australian native fauna (EPBC 1999). Due to this threat to vulnerable wildlife and Australian biodiversity, fox populations are actively controlled. Whilst poison baiting is common and cost-effective in rural areas (Saunders, Gentle, and Dickman 2010), risks to pets and humans restrict control methods in urban areas to trapping and shooting (Marks et al. 1996). These methods are both relatively expensive and difficult to apply at large scales, making urban fox control challenging.

Red foxes are both cryptic and nocturnal, often remaining unnoticed in urban areas despite their high abundance (Phillips and Catling 1991; Doncaster and Macdonald 1997). They thrive on anthropogenic resources and may develop distinct behaviours through urban living (Contesse et al. 2004; Bateman and Fleming 2012; Stepkovitch 2017). Other urban carnivores such as coyotes (*Canis latrans*) display increased boldness and decreased human aversion by comparison to rural counterparts (Bateman and Fleming 2012; Robertson 2018; Breck et al. 2019). Urban carnivores often become larger in size, which may have positive effects on fitness and fecundity (Bateman and Fleming 2012; Stepkovitch et al. 2019). Abundant food can decrease carnivore home ranges, support higher densities, and increase conspecific encounter rates (Bateman and Fleming 2012; Newsome et al. 2015; Dorning and Harris 2019). Urban fox family group sizes are often larger than rural ones, as juvenile females may forego dispersal to assist with cub rearing (Macdonald 1979, 1983; Marks and Bloomfield 1999). Thus, urban environments may enhance conspecific tolerance and social behaviours in foxes (Macdonald 1979, 1983; Marks and Bloomfield 1999; Dorning and Harris 2019).

Although red foxes are known to harbour a diversity of viruses (Bodewes et al. 2013; Lojkić et al. 2016), it is unknown whether urban and rural foxes have different viral compositions. High-density living and increased contact can increase pathogen transmission rates among hosts (Nunn et al. 2015). As such, a high-density population of cryptic urban foxes living in proximity to largely unsuspecting humans could pose an important pathogen risk. Foxes interact with human refuse, including compost and rubbish bins, and consume food scraps from surfaces such as outdoor barbeques and furniture, eat from pet bowls and wildlife feeding stations, and defaecate nearby, increasing the potential for pathogen transfer (Contesse et al. 2004). In addition, as urban animals often habituate to humans (Bateman and Fleming 2012), we might predict an increase in fox–human interactions with the potential for diseased transmission between the two species.

**Figure 1.**
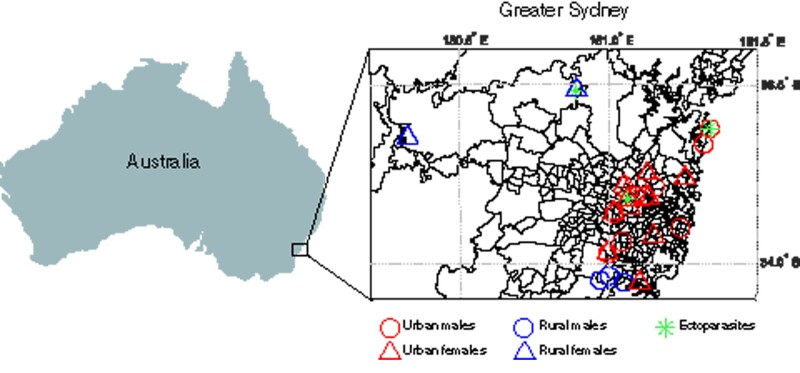
Map of the Greater Sydney region showing fox sampling locations of urban (red) and rural (blue) fox carcases, identified as male (circle) or female (triangle), as well as those harbouring ectoparasites (green asterisk).

Using a meta-transcriptomic approach we describe, for the first time, the virome of the introduced Australian red fox sampled from urban and rural regions. We hypothesised that foxes in urban areas could harbour a greater viral diversity and abundance compared to rural foxes, due to higher population densities and increased conspecific interactions in urban areas. Whilst there is limited information on fox social dynamics in Australia, we also postulated that females could harbour a greater diversity and abundance of viruses than males due to particular social behaviours reported for female foxes in their native ranges, such as cooperative cub rearing (Macdonald 1979, 1983). To this end, samples (liver, faecal, and ectoparasite) were collected from foxes around the Greater Sydney region, Australia, including in urban and rural areas (Fig. 1). Due to diet and organ function, we hypothesised that these tissues comprised very different viromes and together provided a more comprehensive view of the red fox virome. Samples were pooled (based on sampling location, tissue type and sex) and subject to RNA sequencing to reveal viral diversity, evolution, and abundance.

## 2. Materials and methods

### 2.1 Sample collection

The current project was part of a larger research program into urban foxes in partnership with Greater Sydney Local Land Services, a New South Wales State Government organisation responsible for management of pest species across the region. We collected fresh carcases from independent licenced trappers and shooters who were actively controlling foxes in the Greater Sydney region (see Fig. 1 for sample locations). To minimise degradation of RNA, samples were taken as soon as possible after death (03:19:00 ± 02:59:00 h post-mortem, *n* = 27). One carcase had been frozen for approximately 1 week and one carcase had been dead for an unknown amount of time. The foxes used for this study were either trapped in cages and shot, or tracked and shot. One individual was obtained as recent roadkill. Foxes killed by poison baits were excluded.

Whole fox carcases were collected and transported to the laboratory where they were immediately dissected to collect faecal, liver, and ectoparasite samples. All samples were individually stored in RNALater at −80 °C. We sampled a total of twenty-nine individual foxes; thirteen males and sixteen females. For this study, foxes were classified as juvenile if their body mass and body length were less than 3.3 kg and 51 cm, respectively. These values were chosen as the body mass of an adult red fox can range between 3.3 and 8.2 kg, whilst body length can range between 51 and 78 cm (when measured from the tip of the nose to the first vertebra of the tail) (Cavallini 1995). Based on this assessment, twenty-five foxes were classified as adults (twelve males and thirteen females) and four as juveniles (one male and three females).

### 2.2 Sampling in urban and rural areas

Fox sampling relied on coordination with professional pest control operators who focus control efforts in specific locations in accordance with local control initiatives. For this reason, a representative sample across a land-use gradient from urban to rural was not possible. Sufficiently fresh rural and bushland fox samples were also difficult to obtain since poison baiting is the principal control method in these areas. Therefore, ‘rural’ was broadly defined as any natural bushland, national park, mostly agricultural, or sparsely populated region outside the central urban districts, with a human population density of fewer than 500 people per km^2^. Similarly, ‘urban’ was defined as built-up areas inside the central urban district (including parks, gardens, and golf courses) with a population density of more than 500 people per km^2^ either in the area sampled or in the immediate surrounding areas. Human population density information was obtained from the Australian Bureau of Statistics (2016 census data) (Australian Bureau of Statistics 2016a). Central urban districts were defined by the Urban Centres and Localities statistical classification (Australian Bureau of Statistics 2016b). Land-use classification and human population density cut-offs were loosely based on work by Stepkovitch et al. (2019).

### 2.3 RNA extraction and whole-transcriptome sequencing

Qiagen RNeasy Plus Mini Kits were used to extract RNA from liver, faecal, and ectoparasite samples from collected red fox carcases. Thawed samples were transferred to a lysis buffer solution containing 1 per cent β-mercaptoethanol and 0.5 per cent Reagent DX. Samples were homogenised and centrifuged. DNA was removed from the supernatant via gDNA eliminator spin column and RNA was eluted via RNeasy spin column. RNA concentration and purity were measured using the Thermo Fisher Nanodrop. Samples were pooled based on land-use category (urban or rural), sex, and sample type (liver, faecal, or ectoparasite), resulting in nine representative sample pools (Table 1). Adults and juveniles were pooled as only two juveniles were sampled. Ectoparasites included fleas (*Siphonaptera*) and ticks (*Ixodida*). These were not classified below the Order level and due to the small number sampled were also pooled. The TruSeq Stranded Total RNA Ribo-Zero Gold (h/m/r) kit was used to prepare pooled samples for sequencing. Pooled samples were sequenced on the NextSeq 500 with 2× 75 bp output at the Ramaciotti Centre for Genomics at the University of New South Wales, Sydney. Sequencing resulted in nine representative data libraries (Table 1). The raw reads and virus sequences are available on NCBI’s SRA database under BioProject PRJNA640177 GenBank accession numbers MT833874-MT833883.

**Table 1.** Breakdown of red fox representative samples, detailing land use, sex, and sample type, as well as the number of individuals pooled for RNA sequencing.

Representative sample	Land use	Sex	Sample type	Number of individual foxes pooled	Viral transcripts found?
1	Urban	Male	Liver	9	No
2	Urban	Male	Faeces	6	Yes
3	Rural	Male	Liver	3	No
4	Rural	Male	Faeces	3	Yes
5	Urban	Female	Liver	9	No
6	Urban	Female	Faeces	13	Yes
7	Rural	Female	Liver	3	Yes
8	Rural	Female	Faeces	3	Yes
9	Both	Male (1) Female (2)	Ectoparasites	3	Yes

### 2.4 Virus discovery

Sequencing reads were assembled *de novo* into longer sequences (contigs) based on overlapping nucleotide regions using Trinity RNA-Seq (Haas et al. 2013). Assembled contigs were assigned to a taxonomic group (virus, Bacteria, Archaea, and Eukarya) and viruses were identified to their closest species match based on sequence similarity searches against the NCBI nucleotide (nt) and non-redundant protein (nr) databases using BLASTn (Altschul et al. 1990) and Diamond (BLASTX) (Buchfink, Xie, and Huson 2015), respectively. An *e*-value threshold of 1 × 10^−5^ was used as a cut-off to identify positive matches. We removed non-viral hits, including host contigs with similarity to viral sequences (e.g. endogenous viral elements).

### 2.5 Inferring the evolutionary history of fox viruses

We inferred the phylogenetic relationships of the vertebrate-associated viruses identified in the fox samples. Vertebrate-associated viruses were defined as viruses, which shared sequence similarity to other known vertebrate viruses. Due to the high divergence of the virus transcripts, we used only the RNA-dependant RNA polymerase (RdRp) transcripts for phylogenetic analysis. First, the amino acid translations of the viral transcripts were combined with other virus protein sequences from the same virus families obtained from GenBank (Table 2). Second, the sequences were aligned using MAFFT v.3.4, employing the E-INS-I algorithm. Ambiguously aligned regions were removed using trimAl v.1.2 (Capella-Gutiérrez, Silla-Martínez, and Gabaldón 2009). To estimate phylogenetic trees, we selected the optimal model of amino acid substitution identified using the Bayesian Information Criterion as implemented in Modelgenerator v0.85 (Keane et al. 2006) and employed the maximum-likelihood approach available in PhyML v3.1 (Guindon et al. 2010) with 1,000 bootstrap replicates. For the viral transcript matching rabbit haemorrhagic disease virus-2 (RHDV2), we used a nucleotide alignment with similar viruses. New viruses were named after fictional fox characters.

**Table 2.** Vertebrate-associated viral contigs, contig length (nt), percent abundance in their respective pools, and the percent amino acid identity to their closest match on NCBI/GenBank.

Land use (sex)	Virus name (species)	Virus family	Contig length (nt)	% Relative abundance	Closest match (GenBank accession number)	% Amino acid identity	Sample type
Rural (female)	Vixey virus	*Picornaviridae*	2,427	0.007	Canine kobuvirus (AZS64124.1)	97.65	Faeces
	Wilde virus-1	*Picornaviridae*	7,236	5.66	Canine picornavirus (YP_005351240.)	89.18	Faeces
	Wilde virus-3	*Picornaviridae*	1,428	0.0004	Canine picornavirus (AMX81409.1.)	96.22	Liver
	Swiper virus	*Hepeviridae*	7,374	0.01	Elicom virus-1 (YP_009553584.)	28.92	Faeces
	Red fox-associated rabbit haemorrhagic disease virus-2	*Caliciviridae*	7,026	0.14	Rabbit haemorrhagic disease virus-2 (MF421679.1)	99.62	Faeces
Rural (male)	Tod virus-2	*Picornaviridae*	4,263	0.17	Canine picodicistrovirus (YP_007947664.)	98.53	Faeces
	Vulpix virus	*Astroviridae*	2,556	0.046	Feline astrovirus (YP_009052460.)	96.11	Faeces
Urban (female)	Tod virus-1	*Picornaviridae*	2,062	0.0004	Canine picodicistrovirus (YP_007947664.)	98.83	Faeces
	Charmer virus	Picobirnaviridae	448	0.0001	Wolf picobirnavirus (ANS53886.1)	80.27	Faeces
Urban (male)	Wilde virus-2	*Picornaviridae*	1,524	0.00058	Canine picornavirus (YP_005351240.)	73.37	Faeces

### 2.6 Diversity and abundance analysis

Transcript abundance for all viruses (vertebrate and invertebrate associated) was estimated using RSEM within Trinity (Li and Dewey 2011). Specifically, we assessed how many short reads within a given library mapped to a particular transcript. Raw counts were then standardised against the total number of reads within each library. Virome diversity (i.e. virus species richness) and relative abundance were compared among samples using a non-metric multidimensional scaling (nMDS) ordination in conjunction with an analysis of similarities (ANOSIM) based on Bray–Curtis dissimilarity as implemented in the vegan package in R (Oksanen et al. 2019). To determine which viral families were contributing the most to differences between samples, an ‘indicator species’ analysis was performed, using a point biserial coefficient of correlation within the indicspecies package in R (De Cáceres, Legendre, and Moretti 2010).

## 3. Results

**Figure 2.**
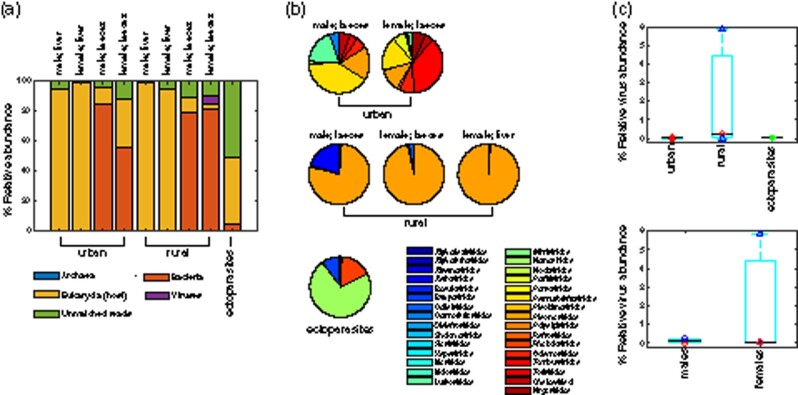
Overview of the red fox virome. (a) Percentage abundance of each taxonomic group identified in each respective pooled sample, standardised against the number of raw reads per pool. Due to their low abundance, archaea (0.002–0.021 per cent) and some of the viral reads (0.001–5.85 per cent) are too small to visualise. (b) Percentage abundance of (eukaryotic-associated) viral families detected in each respective pooled sample (excluding bacteriophage). (c) Boxplots showing percentage abundance of (eukaryotic-associated) viral reads in urban, rural, and ectoparasite samples and males and females. A black line indicates the median and the bottom and top edges of the box indicate the 25th and 75th percentiles, respectively. Raw abundances are superimposed, and the colour and shape of data points are as in Fig. 1.

Meta-transcriptomic sequencing of nine representative pooled samples resulted in 44–57 million paired reads per pool (593,406,706 reads in total). BLAST analyses revealed that the faecal samples were dominated by bacteria (51.17–84.61%), whilst the liver samples were dominated by eukaryotic transcripts (92.90–99.43%), largely comprising fox RNA. Viruses made up a small proportion of the four representative faecal samples (0.002–5.85%) and were detected in only one of the representative liver samples (0.001%). Archaea were detected at very low levels in faecal samples only (0.002–0.021%). The ectoparasites (fleas and ticks) differed substantially to the liver and faecal samples with 50.97 per cent of reads classed as ‘unmatched’ meaning they did not share sequence similarity to any known sequence. The remainder of the contigs from ectoparasite samples were from eukaryotes (44.39%), bacteria (4.64%), and viruses (0.004%). Unmatched reads in liver and faecal samples ranged between 0.52 per cent and 12.22 per cent (Fig. 2a).

Multiple novel vertebrate-associated virus transcripts were identified from both urban and rural foxes, including a hepevirus, picobirnavirus, astrovirus, and various picornaviruses (Table 2). In addition, we found virus transcripts with sequence similarity to RHDV2. Vertebrate-associated virus transcripts represented between 0.4 per cent and 98 per cent of viral reads. The remainder comprised mostly invertebrate-, plant-, and fungi-associated virus transcripts, which were most likely acquired from the foxes’ diet. As no vertebrate-associated viruses were detected in the ectoparasite pool, we performed no further evolutionary analyses.

### 3.1 Virome composition

**Figure 3.**
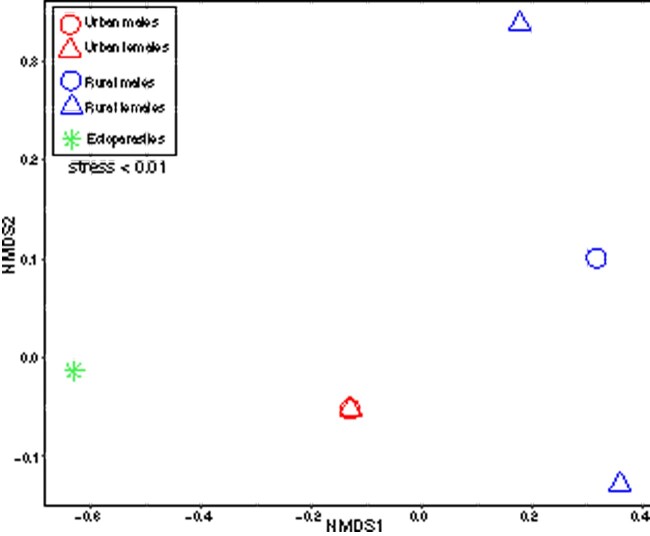
nMDS ordination showing differences in virome composition (at the family level) among samples according to habitat and sex. Individual points represent individual pooled samples. Points closer together have a more similar virome composition (based on Bray–Curtis dissimilarity, which incorporates both the diversity and abundance of viruses) and *vice versa* for those further apart. The stress value was <0.01 and is indicated on the figure.

Urban, rural, and ectoparasite samples had distinctly different virome compositions (ANOSIM *R* = 1, *P *=* *0.0167; Figs 2 and 3). Transcripts from a total of thirty distinct viral families were identified across the six pools in which viral RNA was detected (rural male faeces, rural female faeces, rural female liver, urban male faeces, urban female faeces, and ectoparasites). Overall, twenty-one viral families were identified in transcripts from urban foxes and nineteen from rural foxes. Urban foxes exhibited a slightly higher diversity of viruses compared to rural foxes; transcripts from the latter were heavily dominated by *Picornaviridae*, which made up between 77.33 and 98.97 per cent of the virome of rural foxes (Fig. 2b). Indicator species analysis suggested that *Picornaviridae* were associated with rural samples (stat = 0.978, *P *=* *0.0496), whilst *Nodaviridae* were associated with urban samples (stat = 0.998, *P *=* *0.0498). Viral diversity was higher in females (twenty-five distinct viral families) than in males (thirteen distinct viral families). A much larger percentage of the viral transcripts identified were vertebrate associated in rural foxes (male: 98.23% and female: 97.84%) compared to urban foxes (male: 2.41% and female: 0.39%), although this percentage was higher in males in both groups. In this context, it is important to note that some virus transcripts found here may be the result of contamination by reagents.

On average, total viral abundance (including both vertebrate and non-vertebrate viruses) was higher in rural foxes (2.03 ± 3.31%, *n* = 3) than in urban foxes (0.03 ± 0.04%, *n* = 2), and in female foxes (1.97 ± 3.36%, *n* = 3) than in male foxes (0.12 ± 0.17%, *n* = 2) (Fig. 2c). However, due to the small sample size, differences may be due to some individual animals contributing more to overall abundance or diversity in their respective pool than others. For example, the rural female fox pool (comprising three individual foxes) contained an unusually high number of viruses (>5%) compared to the others. This may have inflated virus abundance counts in females when combined. Whilst virome composition was compared among a relatively small number of samples, this is balanced by the fact that each sample comprises the viromes of multiple individual foxes (*n* = 3–13 foxes per pool; Table 1).

### 3.2 Vertebrate-associated viruses in foxes

#### 3.2.1 Hepeviridae

**Figure 4.**
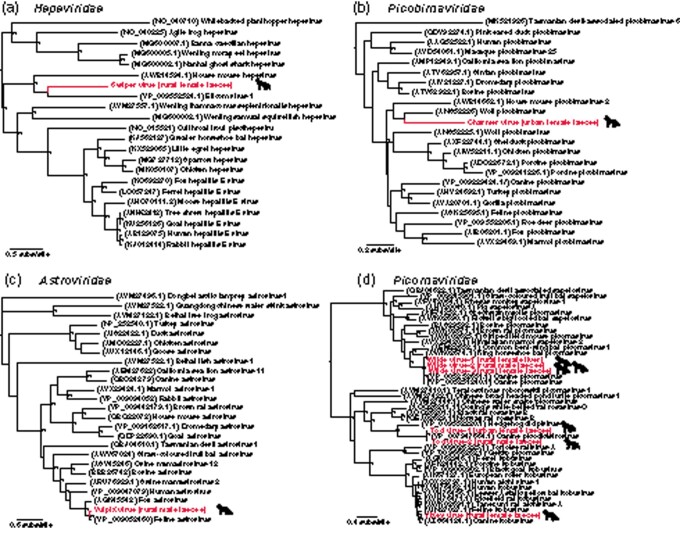
Phylogenetic relationships of likely vertebrate-associated viruses discovered from assembled contigs: (a) *Hepeviridae*, (b) *Picobirnaviridae*, (c) *Astroviridae*, and (d) *Picornaviridae*. The maximum-likelihood phylogenetic trees show the topological position of the newly discovered potential viruses (bold, red text), in the context of their closest relatives. All branches are scaled to the number of amino acid substitutions per site and trees were mid-point rooted for clarity only. An asterisk indicates node support of >70 per cent bootstrap support.

Hepevirus (positive-sense single-stranded RNA viruses) sequences were discovered in the rural female faecal samples. Tentatively named swiper virus, this virus transcript was very distinct in sequence, sharing only 28.92 per cent amino acid identity to its closest relative, elicom virus-1 from mussels, and had a relative abundance of 0.01 per cent (Table 2). Whilst its closest genetic relative is not from a vertebrate host suggesting it may be a diet-associated contaminant, phylogenetic analysis of the RdRp encoding region placed this hepevirus in proximity to both house mouse hepevirus and elicom virus-1, with these viruses forming a distinct monophyletic group (Fig. 4).

#### 3.2.2 Astroviridae

We detected an astrovirus (positive-sense single-stranded RNA virus), tentatively named vulpix virus, in the rural male faecal samples. Notably, the sequence shared a 96.11 per cent amino acid identity with feline astrovirus D1 and had a relative abundance of 0.046 per cent (Table 2). Based on phylogenetic analysis of the RdRp, this virus clustered with other mammalian-associated viruses within the mamastroviruses (Fig. 4).

#### 3.2.3 Picobirnaviridae

Picobirnavirus (double-stranded RNA viruses) sequences were detected in urban male, rural male, and urban female faecal samples. As some of the sequences represented less conserved regions of the viral genome, only one RdRp sequence (from the urban female samples) was used for phylogenetic analysis. The sequence, tentatively named charmer virus, shared an 80.27 per cent amino acid identity with a picobirnavirus found in wolves and had a relative abundance of 0.0001 per cent (Table 2). The sequence also clustered with other mammalian-associated picobirnaviruses (Fig. 4).

#### 3.2.4 Picornaviridae

Several picornaviruses (positive-sense single-stranded RNA viruses) were discovered. Two kobuvirus-related sequences were discovered in the rural female faecal samples. The longer sequence, tentatively named vixey virus, shared highest amino acid identity with canine kobuvirus from a domestic dog (97.65%) and had a relative abundance of 0.007 per cent (Table 2). Analysis of the RdRp region showed that the sequence clustered most closely with feline kobuvirus and other mammalian kobuviruses (Fig. 4).

A number of picodicistrovirus sequences were detected in the urban male, rural male, and urban female faecal samples. Two of the sequences, tentatively named tod virus-1 and tod virus-2, both shared 98 per cent amino acid identity with canine picodicistrovirus (Table 2). Based on analysis of the RdRp region, the sequences clustered together with mammalian dicipivirus and rosaviruses as well as reptilian picornaviruses (Fig. 4).

Multiple picornavirus sequences were identified in the rural male faecal and the rural female faecal and liver samples. Three sequences, tentatively named wilde virus-1, 2, and 3, all shared between 73 and 89 per cent amino acid identity with canine picornavirus and had relative abundances of 5.66 per cent, 0.00058 per cent, and 0.0004 per cent, respectively (Table 2). These sequences clustered with other mammalian picornaviruses (Fig. 4).

#### 3.2.5 Caliciviridae

**Figure 5.**
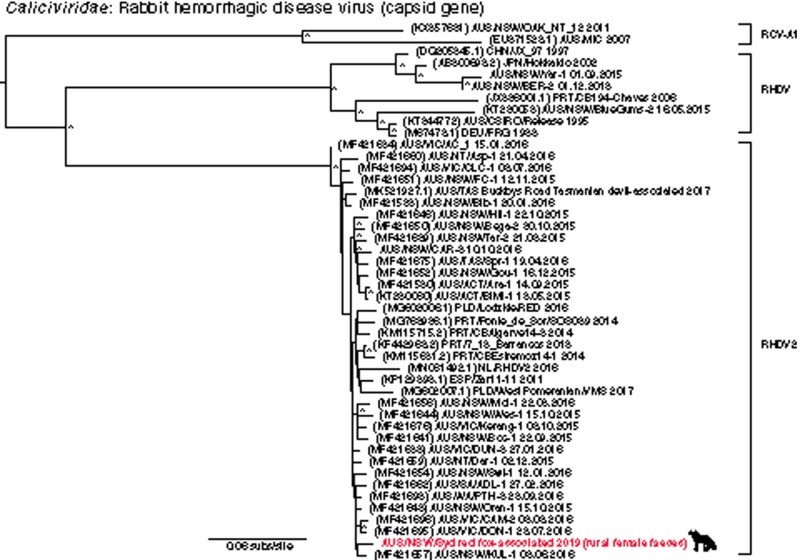
A maximum-likelihood phylogenetic tree showing the topological position of RHDV2 capsid gene in the red fox (bold, red text), in the context of its closest relatives. Major clades are labelled. All branches are scaled to the number of nucleotide substitutions per site and trees were mid-point rooted for clarity only. An asterisk indicates node support of >70 per cent bootstrap support.

One of the most striking observations was the identification of RHDV2 (a positive-sense single-stranded RNA virus) in rural female and urban male faecal samples. The viral sequence in the rural female samples shared a 99.62 per cent amino acid identity with RHDV2 isolated from rabbits between 2015 and 2016 and had a relative abundance of 0.14 per cent (Table 2) (Fig. 5). The viral sequence in the urban male samples was too short to enable phylogenetic analysis. This is the second time that RHDV2 has been found in non-rabbit hosts (Chong et al. 2019), presumably through rabbit consumption in this case.

## 4. Discussion

We show that Sydney’s red foxes, in both urban and rural environments, harbour a wide diversity of viruses, some of which are genetically similar to those that infect domestic pets and humans. Domestic mammals tend to hold central positions in mammal viral transmission networks (Wells et al. 2020). The close genetic similarity of the viruses found here to viruses frequently found in common domestic pets such as cats and dogs suggests that cross-species transmission between foxes and domestic species may have occurred. The most cited case of viral transmission between humans and domestic pets is the transmission of rabies virus (Ghasemzadeh and Namazi 2015), although other examples include noroviruses from dogs, isolated cases of influenza A(H7N2) virus from cats (Lee et al. 2017; Marinova-Petkova et al. 2017), and numerous bacterial diseases and parasites (Ghasemzadeh and Namazi 2015; O’Neil 2018). There may also be additional cases of viral sharing between humans and their pets, although these may go undiagnosed due to insufficient knowledge of the genetic variability of these viruses and their relationships with hosts.

All vertebrate-associated viruses found here were RNA viruses. Although this may in part be due to the reliance on transcript-based viral detection, RNA viruses are in general characterised by lower host specificity than DNA viruses, reflecting an increased occurrence of cross-species transmission (Geoghegan, Duchêne, and Holmes 2017; Wells et al. 2020). The opportunity for interactions between urban wildlife, pets, and humans provides likely transmission pathways for novel RNA viruses. Indeed, eukaryotic parasites are already known to infect human hosts following the wildlife–domestic pet–human transmission network (Wells et al. 2018).

We discovered viral transcripts with some sequence similarity to the *Hepeviridae* that cause hepatitis E in mammals, which has already been isolated from various domestic and wild animals including foxes in the Netherlands (Meng 2010; Bodewes et al. 2013). Confirmed zoonotic cases include transmission to humans from domestic pigs, cats, and wild rodents (Meng 2010; Dremsek et al. 2012). In contrast, the hepevirus detected here was phylogenetically distinct from the fox hepatitis E virus previously detected (Bodewes et al. 2013) and instead was more closely related to hepeviruses detected in freshwater mussels and a house mouse. Hence, although we have classed the virus as vertebrate associated, its divergent phylogenetic position could in fact mean that it results from dietary consumption.

The astrovirus transcript (vulpix virus) showed the greatest sequence similarity (96 per cent) to astroviruses from domestic cats as well as from other foxes, humans, and pigs. Astroviruses have a broad host range (Donato and Vijaykrishna 2017) and are frequently detected in the faeces of mammals, birds, and humans with gastroenteritis (Finkbeiner et al. 2009; De Benedictis et al. 2011). Astroviruses have also been associated with other diseases and disorders such as shaking syndrome in minks (Blomström et al. 2010), neurological disease in cattle (Li et al. 2013), and encephalitis in humans (Quan et al. 2010). Some human astroviruses are more closely related to those in animals than to each other, suggesting that these viruses periodically emerge from zoonotic origins (Kapoor et al. 2009). The similarity of fox astroviruses to those found in cats indicates that these viruses may have jumped hosts in the past and highlights further the potential role of domestic pets and wildlife in virus transmission.

Picobirnaviruses are found in humans and other mammals and are thought to be linked with gastroenteritis, however their role in disease remains unclear (Malik et al. 2014; Conceição-Neto et al. 2016). The picobirnavirus-related transcript found here showed the greatest sequence similarly to a picobirnavirus found in wolves with diarrhoeic symptoms (Conceição-Neto et al. 2016). It is also similar to picobirnaviruses described as potentially zoonotic in humans with gastroenteritis (Yinda et al. 2019). There is, however, evidence that picobirnaviruses may actually be bacteriophage rather than eukaryote-associated viruses (Krishnamurthy and Wang 2018), such that the virology of these viruses is currently unclear.

We identified novel fox viruses within the *Picornaviridae* belonging to three distinct genera: kobuvirus, picodicistrovirus, and picornavirus. The *Picornaviridae* are a large and diverse family that include viruses associated with a variety of human diseases such as hand, foot and mouth disease, polio, myocarditis, hepatitis A virus, and rhinovirus (Zell 2018). All viral sequences here were most closely related to those viruses previously found in dogs. Whilst we cannot assume that these viruses cause disease, kobuviruses have been isolated from dogs and other mammals with diarrhoeic symptoms (Reuter, Boros, and Pankovics 2011; Di Martino et al. 2013). Additionally, the fox picornaviruses found here are closely related to sapeloviruses that cause encephalitis in domestic pigs (Lan et al. 2011; Schock et al. 2014; Arruda et al. 2017).

Finally, and of particular note, we identified RHDV2 in fox faeces. RHDV was initially released (or escaped) in Australia in 1995 following testing as a biological control agent for invasive rabbits. A novel variant of the disease, RHDV2, began circulating in Australia in 2015 and is presumed to be an incursion from Europe where it first emerged in 2010 (Hall et al. 2015). RHDV2 has become the dominant strain circulating in Australia’s wild rabbits (Mahar et al. 2018). The virus identified here was most closely related to RHDV2 strains found in rabbits in New South Wales, Australia in 2015–6. It is likely, then, that Sydney foxes consume diseased rabbits and the virus is simply a gut contaminant with no active RHDV2 replication in the fox host. Although it is worth noting that antibodies against RHDV have been detected in red foxes in Germany, there was no evidence of illness or viral replication (Frölich, Klima, and Dedek 1998).

Urbanisation influences pathogen exposure and prevalence in wildlife. For example, the prevalence of parvovirus increases with proximity to urban areas in grey foxes (*Urocyon cinereoargenteus*) in the USA (Riley, Foley, and Chomel 2004), and dogs in urban areas in Brazil harbour more tick-borne pathogens than rural dogs (Vieira et al. 2013). In addition, the prevalence of West Nile virus in wild birds in the USA increases with proximity to urban areas and human population density (Gibbs et al. 2006). Here, we found the highest overall viral abundance in rural foxes whilst urban foxes harboured a slightly higher diversity of viruses (Fig. 2b and c). Whilst differences in overall abundance and diversity of viruses present in foxes may be a reflection of differences in diet and environment, we found rural foxes to have a much higher abundance of vertebrate-associated viruses than urban. It has previously been suggested that red foxes in highly urbanised areas experience lower exposure to canine distemper virus due to reduced movement opportunities as a result of wildlife corridors being absent in densely built-up areas (Gras et al. 2018). By comparison, exposure to canine distemper virus increased in areas with more natural habitats (Gras et al. 2018).

It is possible that urban living reduces fox susceptibility to viral infection by positively influencing host immunity. For example, an abundance of rich food sources would increase nutritional intake, positively influencing overall health and condition and hence resistance to viral infections (Beldomenico and Begon 2010). Kit foxes (*Vulpes macrotis*) in urban areas in California show less nutritional stress, increased body condition, and improved immune function when compared to foxes in a nearby nature reserve (Cypher and Frost 1999). Australian lace monitors (*Varanus varius*) consuming human refuse experience improved body condition and reduced blood parasite infection compared to those that do not subsist on anthropogenic food waste (Jessop et al. 2012). Foxes in urban Sydney grow larger and are heavier than foxes in rural areas (Stepkovitch et al. 2019), and there may be an advantage to consuming anthropogenic food sources for overall condition and pathogen resistance.

Across both rural and urban habitats, we observed that female foxes harboured a higher abundance and had almost twice the diversity of viruses found in male foxes (when including both vertebrate and non-vertebrate associated). This difference in viromes may indicate different ecologies and behaviours in male and female foxes. Whilst other studies looking at sex differences and immunity suggest that females typically display stronger immune responses and reduced pathogen load compared to males (Klein 2000), greater sociality in females (Macdonald 1979, 1983) may increase viral transmission opportunities. However, our understanding of red fox sociality in Australia is limited (Newsome 1995) and males may be more likely to be involved in aggressive encounters with conspecifics than females (White and Harris 1994). Alternatively, a combination of biological and ecological differences, such as hormones, diet, and environment, could contribute to variation in male and female viromes (Vemuri et al. 2019).

Multiple co-occurring factors could simultaneously affect viral infection in Sydney’s foxes. Additional assessments of habitat structure, fox densities, movement behaviours, and social dynamics in urban and rural areas in the Greater Sydney region will help to elucidate such factors. An obvious extension to this work is to examine fox viromes across a more comprehensive urban-rural gradient, including foxes from more isolated bush habitats. This would help us to understand differences in pathogen prevalence and transmission between isolated natural habitats and more disturbed environments, and how introduced species such as foxes contribute to disease prevalence across different ecosystems. Another useful approach could compare viral transmission dynamics in red foxes between their native and introduced ranges.

Human encroachment on wild environments and the adaptation of wild animals to urban areas continues to intensify human–wildlife interactions. The effects of urbanisation on wildlife pathogen dynamics may have unexpected consequences for human and domestic animal health. Although we cannot say definitively that the viruses identified here cause disease outbreaks or spill-over events, it is clear that foxes living in Greater Sydney carry viruses that are related to those found in domestic animals and humans. Our findings indicate that foxes may be reservoirs for viral pathogens with zoonotic potential.

Conflict of interest: None declared.
